# Machine Learning Applicability for Classification of PAD/VCD Chemotherapy Response Using 53 Multiple Myeloma RNA Sequencing Profiles

**DOI:** 10.3389/fonc.2021.652063

**Published:** 2021-04-15

**Authors:** Nicolas Borisov, Anna Sergeeva, Maria Suntsova, Mikhail Raevskiy, Nurshat Gaifullin, Larisa Mendeleeva, Alexander Gudkov, Maria Nareiko, Andrew Garazha, Victor Tkachev, Xinmin Li, Maxim Sorokin, Vadim Surin, Anton Buzdin

**Affiliations:** ^1^ Moscow Institute of Physics and Technology, Laboratory for Translational Genomic Bioinformatics, Dolgoprudny, Russia; ^2^ National Research Center for Hematology, Ministry of Health of the Russian Federation, Moscow, Russia; ^3^ I.M. Sechenov First Moscow State Medical University, Institute of Personalized Medicine, Moscow, Russia; ^4^ Shemyakin-Ovchinnikov Institute of Bioorganic Chemistry, Group for Genomic Analysis of Cell Signaling Systems, Moscow, Russia; ^5^ Department of Pathology, Faculty of Medicine, Lomonosov Moscow State University, Moscow, Russia; ^6^ Omicsway Corp., Research Department, Walnut, CA, United States; ^7^ Oncobox Ltd., Research Department, Moscow, Russia; ^8^ Department of Pathology and Laboratory Medicine, University of California Los Angeles, Los Angeles, CA, United States

**Keywords:** multiple myeloma, bortezomib, gene expression, machine learning, treatment response, fibroblast growth factor receptor 3, PAD, VCD

## Abstract

Multiple myeloma (MM) affects ~500,000 people and results in ~100,000 deaths annually, being currently considered treatable but incurable. There are several MM chemotherapy treatment regimens, among which eleven include bortezomib, a proteasome-targeted drug. MM patients respond differently to bortezomib, and new prognostic biomarkers are needed to personalize treatments. However, there is a shortage of clinically annotated MM molecular data that could be used to establish novel molecular diagnostics. We report new RNA sequencing profiles for 53 MM patients annotated with responses on two similar chemotherapy regimens: bortezomib, doxorubicin, dexamethasone (PAD), and bortezomib, cyclophosphamide, dexamethasone (VCD), or with responses to their combinations. Fourteen patients received both PAD and VCD; six received only PAD, and 33 received only VCD. We compared profiles for the good and poor responders and found five genes commonly regulated here and in the previous datasets for other bortezomib regimens (all upregulated in the good responders): *FGFR3*, *MAF*, *IGHA2*, *IGHV1-69*, and *GRB14*. Four of these genes are linked with known immunoglobulin locus rearrangements. We then used five machine learning (ML) methods to build a classifier distinguishing good and poor responders for two cohorts: PAD + VCD (53 patients), and separately VCD (47 patients). We showed that the application of FloWPS dynamic data trimming was beneficial for all ML methods tested in both cohorts, and also in the previous MM bortezomib datasets. However, the ML models build for the different datasets did not allow cross-transferring, which can be due to different treatment regimens, experimental profiling methods, and MM heterogeneity.

## Introduction

Multiple myeloma (MM) is a hematological cancer which arises from abnormal antibody producing white blood plasma cells (1). MM affects approximately 500,000 people and results in ~100,000 deaths annually (2, 3) being currently considered treatable but rarely curable (4, 5). There are several MM chemotherapy treatment regimens currently in use, among which eleven include bortezomib (6) (**Table 1**). Bortezomib is a targeted drug that specifically binds and inhibits 26S proteasome, thus affecting proteolytic degradation pathways (20). Patients with MM respond differently on bortezomib-containing treatment schemes (21), and many patients develop adverse effects including neuromuscular and cardiovascular toxicity (22). Thus, new prognostic biomarkers are needed to personalize treatments with bortezomib (21).

**Table 1:** Bortezomib containing chemotherapy regimens currently in use for the first-line treatment of multiple myeloma.

Chemotherapy regimen	Primary therapy for transplant candidates	Primary therapy for non-transplant candidates
Bortezomib + Doxorubicin + Dexamethasone (PAD)	yes (7)	no
Bortezomib + Cyclophosphamide + Dexamethasone (VCD)	yes (8, 9)	yes (8, 10)
Bortezomib + Lenalidomide + Dexamethasone	yes (11)	yes (12)
Daratumumab + Bortezomib + Lenalidomide + Dexamethasone	yes (13)	no
Bortezomib + Thalidomide + Dexamethasone	yes (14)	no
Daratumumab + Bortezomib + Cyclophosphamide + Dexamethasone	yes (15)	no
Daratumumab + Bortezomib + Thalidomide + Dexamethasone	yes (16)	no
Bortezomib + Thalidomide + Dexamethasone + Cisplatin + Doxorubicin + Cyclophosphamide + Etoposide (VTD-PACE)	yes (17)	no
Daratumumab + Bortezomib + Melphalan + Prednisone	no	yes (18)
Daratumumab + Bortezomib + Cyclophosphamide + Dexamethasone	no	yes (15)
Bortezomib + Dexamethasone	no	yes (19)

High-throughput gene expression data including RNA sequencing profiles can be used for finding effective cancer biomarkers (23, 24). There is a shortage now for clinically annotated molecular profiles of MM that could be used to establish novel molecular diagnostics for most of the current clinical treatment regimens. For several regimens with bortezomib, MM gene expression profiles had been previously established and published for patients who were classified as either responders or non-responders. For example, in a study (25) using Affymetrix Human Genome U133 expression microarrays, 169 MM profiles were published for 85 responder patients and for 84 non-responders on monotherapy with bortezomib (26). In another paper (27) an Affymetrix Human Exon 1.0 ST Array expression dataset was published with the 33 responder and 28 non-responder profiles for the bortezomib monotherapy followed by autologous stem cell transplantation (ASCT) (28). However, monotherapy with bortezomib is not currently a recommended option for the treatment of MM due to its lower efficacy compared to combinational therapies (6). For one of the options currently in clinical use for the MM namely bortezomib + thalidomide + dexamethasone scheme, there is a publicly available dataset (29) obtained using Affymetrix Human Genome U133 Plus arrays for the 69 responder and 49 non-responder patients (28). Other examples account for the studies of bortezomib, doxorubicin, and dexamethasone (PAD) chemotherapy regimen at Myeloma Institute for Research and Therapy (55 responders and 153 non-responders) (30–40), and during Dutch-Belgian HOVON project (30–32, 41–44), where 94 responders and 59 non-responders were investigated; for both studies Affymetrix microarrays were used.

In this study we report new RNA sequencing profiles for 58 (53 after mapped reads threshold filtering) MM patients annotated with the documented responses on two chemotherapy regimens that include bortezomib: PAD, or bortezomib, cyclophosphamide, and dexamethasone (VCD). These regimens are similar in their composition and differ in the presence of doxorubicin that interferes with the DNA replication by intercalating with the nucleobases (45) or cyclophosphamide that produces crosslinks between the DNA strands (46). Both treatment regimens showed clinical benefit and were accepted as first-line treatment of multiple myeloma internationally and in the Russian Federation (**Table 1**). To our knowledge, this is the first annotated RNA sequencing molecular dataset for the PAD and VCD regimens of MM chemotherapy. In addition, the current profiles were obtained using the same protocols, equipment and reagents as for the ANTE database of RNA sequencing profiles for healthy human tissues and are, therefore, fully compatible with the enclosed eleven profiles for the normal CD138+ cells (47).

The MM biosamples investigated here were taken prior to the first-line chemotherapy treatments and subjected to RNA sequencing. Following treatment, the patients were clinically characterized to assess clinical responses according to the International Myeloma Working Group. Totally, 11 high-quality profiles were obtained for the “complete responders” (CR), 17 for “very good partial responders” (VGPR), 12 for “partial responders” (PR), and 13 for “minimal responders” (MR), where CR + VGPR can be considered good responders and PR + MR—poor responders. Among them, 14 patients received both PAD and VCD treatments (3–12 courses, sequentially), 33 received only VCD (3–12 courses) and 6—only PAD (4–6 courses).

We then used enhanced algorithms for five machine learning (ML) methods to build a classifier distinguishing good and poor treatment responders: support vector machines (SVM), Tikhonov (ridge) regression (RR), binomial naïve Bayes (BNB), random forest (RF) and multi-layer perceptron (MLP). The best result for full PAD+VCD cohort (n = 53) was produced by BNB method (AUC 0.84, sensitivity >0.8, specificity >0.84), and for the VCD cohort (n = 47) by the MLP method (AUC 0.89, sensitivity >0.87, specificity >0.83). In both optimal solutions, FloWPS dynamic data trimming method (26, 48, 49) was used to reduce data dimensionality. We also showed that the same approach was effective for classifying other annotated MM datasets with different bortezomib treatment regimens. We also compared gene expression profiles for the good and poor responders and found five genes commonly regulated here and in the previous datasets (all upregulated in the good responders): *FGFR3*, *MAF*, *IGHA2*, *IGHV1-69*, and *GRB14*.

## Materials and Methods

### Clinically Annotated Biosamples

From March 2016 till June 2018, we collected 58 biosamples of bone marrow cells enriched for the presence of CD138-expressing mononuclear cells, isolated for the patients diagnosed with multiple myeloma (MM) and prescribed with further first-line chemotherapy treatments according to PAD and/or VCD regimens. The MM patients were 29–78 years old, mean age was 58 y.o., 31 male and 27 female patients (**Supplementary Table 1**). To isolate mononuclear cells, we used Ficoll Paque Plus medium (Sigma) according to the manufacturer’s recommendations. CD138+ cells fractions were obtained using magnetic granules coated with CD138-specific human antibodies MicroBeads (Miltenyi Biotec) and MS Columns (Miltenyi Biotec), according to the manufacturer’s recommendations. Cells were counted by Scepter™ 2.0 Handheld Automated Cell Counter (Merck Millipore) and immediately subjected to RNA extraction.

In parallel, a set of normal samples of CD138+ mononuclear cells was isolated from eleven 25–42 y.o. (mean age 32 y.o.; five males and six females) healthy volunteers as described in (47).

In all tumor related CD138+ experimental fractions the content of MM cells varied between 45 and 97%, as estimated by the pathologist using BD FACSCanto II flow cytometer (Becton Dickinson, USA) and phycoerythrin-conjugated anti-CD138 antibodies. This fraction was then subjected to RNA sequencing with approximately 30 million sequencing reads per library. In parallel, the patient treatment responses on bortezomib, doxorubicin, and dexamethasone (PAD) or bortezomib, cyclophosphamide, and dexamethasone (VCD) regimens, or their combinations, were registered and documented. Among them, 17 patients received both PAD and VCD treatments (3–12 courses, sequentially), 36 received only VCD (3–12 courses), and 5-only PAD (4–6 courses). Totally, 13 RNA sequencing profiles were obtained for the “complete responders” (CR), 17 for “very good partial responders” (VGPR), 14 for “partial responders” (PR), and 16 for “minimal responders” (MR), **Supplementary Table 1**. Moreover, for two poor responder cases (patients 111 and 115) we isolated MM mononuclear CD138+ cells following tumor relapse on PAD + VCD treatment and performed RNA sequencing (**Supplementary Table 1**).

For all the biosamples, informed written consents to participate in this study were collected from the patient’s legal representatives. The consent procedure and the design of the study were approved by the ethical committees of the Sechenov Moscow First Medical University, of the Clinical Center Vitamed (Moscow), and of the National Research Center for Hematology (Moscow, Russia).

### Preparation of Libraries and RNA Sequencing

For RNA extraction, cells were resuspended in TRI Reagent (MRC) and then Direct-zol RNA MiniPrep (Zymo Research) was used for the RNA extraction. RNA was quantified using Nanodrop (Thermo Fisher Scientific), ethanol-precipitated, and stored in liquid nitrogen until sequencing. For library preparation, RNA Integrity Number (RIN) was measured using Agilent 2100 bioanalyzer. Agilent RNA 6000 Nano or Qubit RNA Assay Kits were used to measure RNA concentration. For depletion of ribosomal RNA, we used KAPA RNA Hyper with RiboErase Kit (KAPA Biosystems). Different adaptors were used for multiplexing samples in one sequencing run. Library concentrations and quality were measured using Qubit ds DNA HS Assay kit (Life Technologies) and Agilent Tapestation (Agilent). RNA sequencing was performed using Illumina HiSeq 3000 equipment for single end sequencing, 50 bp read length, for approximately 30 million raw reads per sample. Data quality check was conducted using Illumina SAV. De-multiplexing was performed using Illumina Bcl2fastq2 v 2.17 software. In parallel, we also isolated fractions of control CD138+ cells from eleven healthy volunteers and subjected them to RNA sequencing using the same protocol, equipment and reagents. The healthy donor profiles were published previously as part of the ANTE atlas of RNA sequencing data in healthy tissues (47).

### Processing of RNA Sequencing Data

RNA sequencing FASTQ files were processed with STAR aligner (27) in ‘GeneCounts’ mode with the Ensembl human transcriptome annotation (Build version GRCh38 and transcript annotation GRCh38.89). Ensembl gene IDs were converted to HGNC gene symbols using Complete HGNC dataset (https://www.genenames.org, database version of July 13, 2017. In total, expression levels were established for 36,596 annotated genes with corresponding HGNC identifiers. Additional quality control (QC) metrics for obtained data were generated using NCBI MAGIC software (28, 49, 50). All metrics and detailed protocol for each sample can be found in **Supplementary Table 2**.

### Data Clustering

‘1’ was added to all raw gene counts prior to cluster analyses, to avoid zero expression values, as described by Dillies et al. (51), the gene expression data were merged into single datasets and quantile normalized (52). Hierarchical clustering was performed using R ward.D2 method. The dendrogram was visualized using custom R script.

### Dataset Preparation for Machine Learning (ML) Applications

According to (26, 48, 49), the preparation of datasets for the analysis included several steps: (i) normalization of expression levels using the DESeq2 method (51); (ii) finding top 30 marker genes having the highest AUC values for discriminating good and poor responder cases; (iii) performing the leave-one-out (LOO) cross-validation procedure to identify robust core marker gene set that will be used for building the ML models. The latter core marker gene set is an intersection of top 30 marker gene sets for all combinations of but one samples (26, 28, 48, 49).

### ML Applications

Although modern ML applications in clinical cancer genomics may rely on deep learning methods (53–55), they require large preceding case cohorts (56), which was not the case for neither of the MM expression datasets under investigation. Thus, to further characterize them we used several non-deep ML methods implemented in the Python *sklearn* library (56).

The ML analysis of the experimental MM profiles was performed in two modes. First—when all 53 patients were included whose gene expression profiles passed the quality control (PAD+VCD cohort). Second, when 47 patients were included who had either only VCD or combination of PAD and VCD, but not only PAD (VCD cohort).

For each ML method we used a data trimming/preprocessing step using FloWPS method (R package flowpspkg.tar.gz) to increase robustness and efficiency due to individual sample-specific selection of training dataset (26, 48, 49). Among the ML methods, we used linear support vector machines (SVM) and ridge regression (RR) with default parameter settings for the *sklearn* package. Additionally, we applied random forest (RF), binomial naïve Bayes (BNB), and multi-layer perceptron (MLP) with the settings, which previously showed the best performance for building cancer responder classifiers (26). For RF these settings were *n_estimators* = 30, *criterion* = ‘entropy’. For BNB: *alpha* = 1.0, *binarize* = 0.0, and *fit_prior* = False. For MLP: *hidden_layer_sizes* = 30, *alpha* = 0.001. To compensate possible effect of unequal number of responder and non-responder samples, all SVM and RF calculations were done with setting *class_weight =* ‘balanced’ and *class_weight = *’balanced_subsample’, respectively. All other parameters were used with the default settings.

### Data Records

MM gene expression profiles were deposited to Gene Expression Omnibus database (GEO) under accession number GSE159426. The data is provided as a matrix of raw counts as produced by STAR. The mapping statistic for the corresponding dataset is shown in **Supplementary Table 2**. The RNA sequencing profiles for healthy CD138-positive controls were deposited in GEO database with accession number GSE120795.

### Code Availability

R code for building dendrograms with bar plots is freely available on Gitlab at: https://gitlab.com/oncobox/watermelon_multisection/blob/master/utils/gallow_plot.R. Flowpspkg.tar.gz is available on Gitlab at: https://gitlab.com/borisov_oncobox/flowpspkg.

## Results and Discussion

### Initial Analysis of RNA Sequencing Data

**Figure 1:**
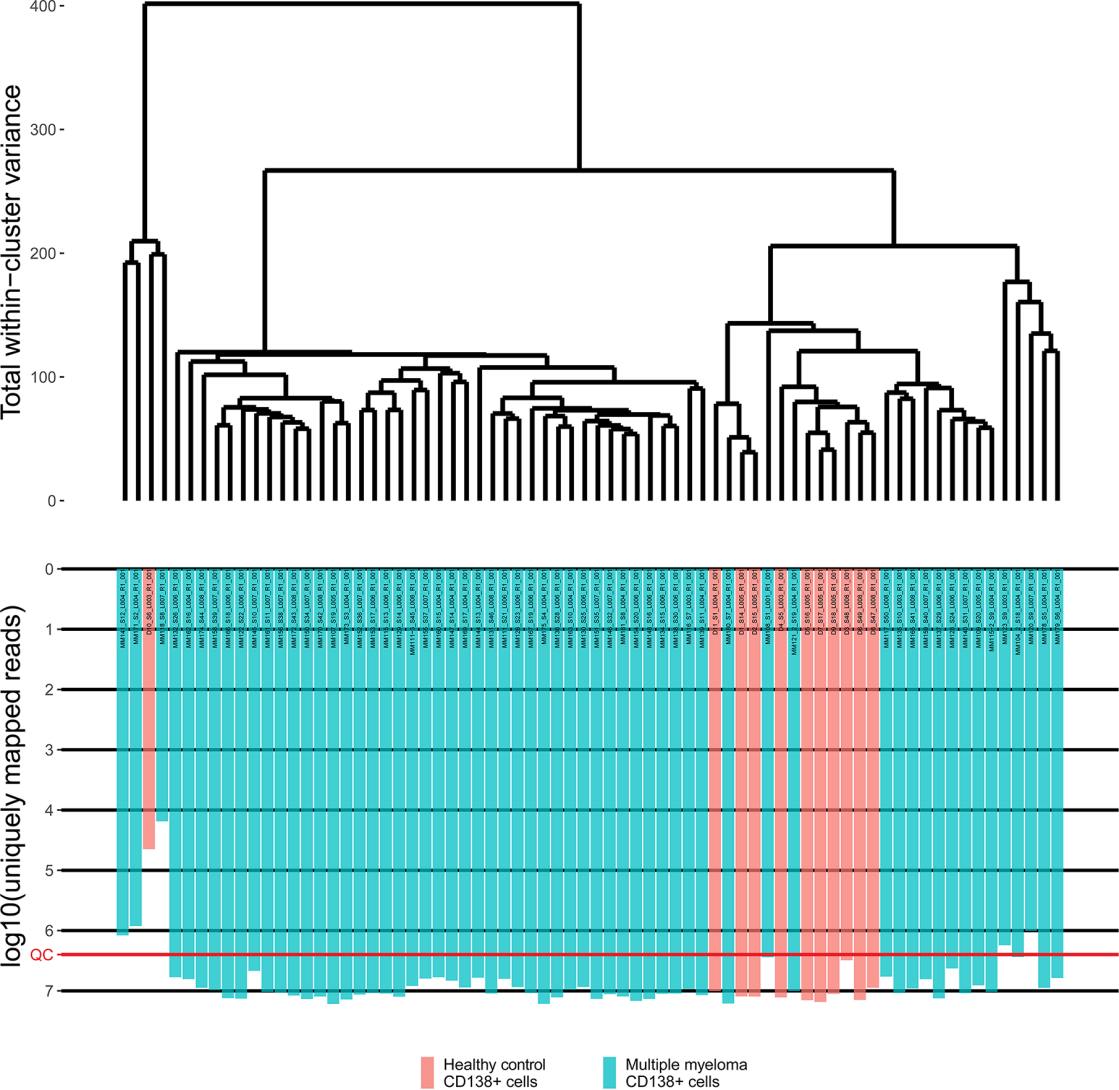
The hierarchical clustering dendrogram of all experimental RNA sequencing profiles of the control and multiple myeloma samples. Gene expression data were used to calculate Euclidian distances between the samples. Color indicates the sample type. The lower scale indicates the number of uniquely mapped reads. QC denotes the quality control threshold of 2.5 million uniquely mapped reads.

**Figure 2:**
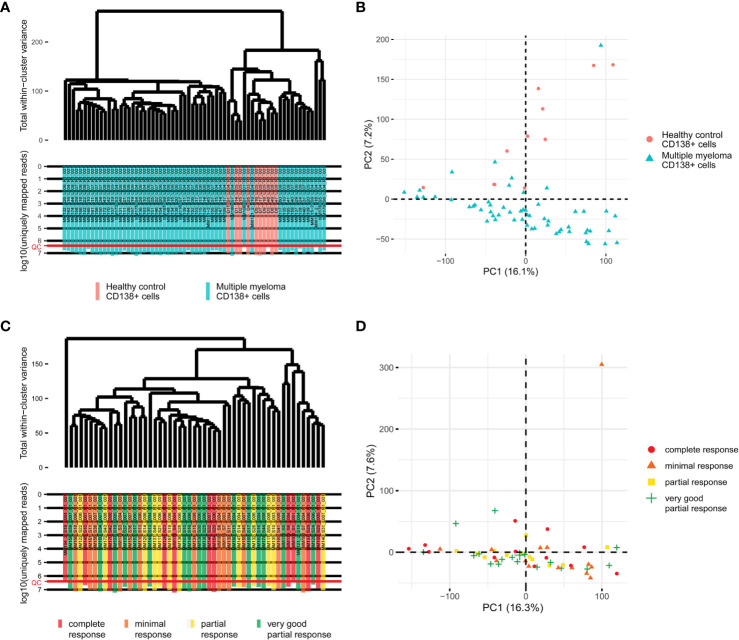
**(A)** The hierarchical clustering dendrogram of QC-passed experimental RNA sequencing profiles of the control and multiple myeloma samples. Gene expression data were used to calculate Euclidian distances between the samples. The color markers indicate the sample types. The lower scale indicates the number of uniquely mapped reads. ‘QC’ denotes the quality control threshold of 2.5 million uniquely mapped reads. **(B)** PCA for QC-passed experimental RNA sequencing profiles of the control and multiple myeloma samples. The color markers indicate the sample types. **(C)** The hierarchical clustering dendrogram of QC-passed experimental RNA sequencing profiles of the multiple myeloma samples. Gene expression data were used to calculate Euclidian distances between the samples. The color markers indicate the response. The lower scale indicates the number of uniquely mapped reads. ‘QC’ denotes the quality control threshold of 2.5 million uniquely mapped reads. **(D)** PCA for QC-passed experimental RNA sequencing profiles of the multiple myeloma samples. The color markers indicate the response.

Primary RNA sequencing data were characterized in detail with the NCBI MAGIC software (57) (**Supplementary Table 2**) and analyzed to assess if the profiles obtained are congruent with the biological nature of the biosamples under study. To this end we mixed the MM data obtained here with the profiles obtained by us using the same protocols, equipment and reagents for eleven samples of CD138+ cells of healthy volunteers (47). We performed hierarchical clustering analysis and observed that in line with the biological significance with one exception the norms formed a compact cluster on the dendrogram separately from the cancers (**Figure 1**). Furthermore, the outstanding normal profile had relatively low number of sequencing reads (**Figure 1**) and didn’t meet the previously established quality control (QC) criterion for this RNA sequencing protocol of having at least 2.5 million uniquely gene-mapped reads per library (47). This established threshold effectively marked samples with low quality values of other QC metrics, e.g. proportion of genomic counts, high rate of mismatches, number of reads spanning splice junction, high percentage of ribosomal counts (47). Filtering of the profiles that didn’t meet mapped-reads QC resulted in a tight clustering both on the dendrogram and on the principal component analysis (PCA) plot and removed healthy outlier (**Figures 2A, B** **)**. However, the good (CR + VGPR) and poor (PR + MR) MM responders showed mixed trend and didn’t form any response-specific clusters (**Figures 2C, D** **)**.

### Building of ML-Assisted Classifiers for VCD MM Responders and Non-Responders

For our further analyses we used molecular profiles that passed mapped-reads QC and represented 53 MM patients (**Supplementary Table 1**), where 28 were classified as the good (CR + VGPR) and 25 as the poor (MR + PR) responders.

Reducing data dimensionality in disproportionately rich datasets is required for statistically justified tests (49). Prior to using machine learning (ML) approaches, we performed feature selection procedure to identify core marker gene expression set comparable in size to the number of the patient cases under analysis (26, 28). To this end we selected the most informative fraction of the initial data that can distinguish between the good and poor treatment responder classes using a leave-one-out-based method (48). Because of the size of our MM dataset (n = 53 for full cohort and 47 for VCD) the n-fold cross-validation scheme is too rough for such a limited dataset, and the leave-one-out (i.e., 53/47-fold cross-validation approach for full/VCD cohort, respectively) is the best way to obtain more accurate results.

For each clinical case *i = 1, … 53/47*, we determined the top 30 marker genes that distinguished responder and non-responder cases in a sub-dataset that contains all samples but *i*. For all 52/46 such sub-datasets each having 52/46 cases, we interrogated each gene taken one by one and obtained the set of top 30 genes showing the highest ROC AUC values for the difference between responder and non-responder profiles. Area under the ROC curve (AUC) is the universal metric of a biomarker robustness depending on its sensitivity and specificity (28). It varies from 0.5 till 1, and the standard discrimination threshold is typically set as 0.7, where the items with greater AUC are thought high-quality biomarkers (58). AUC is broadly used for scoring of molecular biomarkers in oncology (23, 59–62).

**Figure 3:**
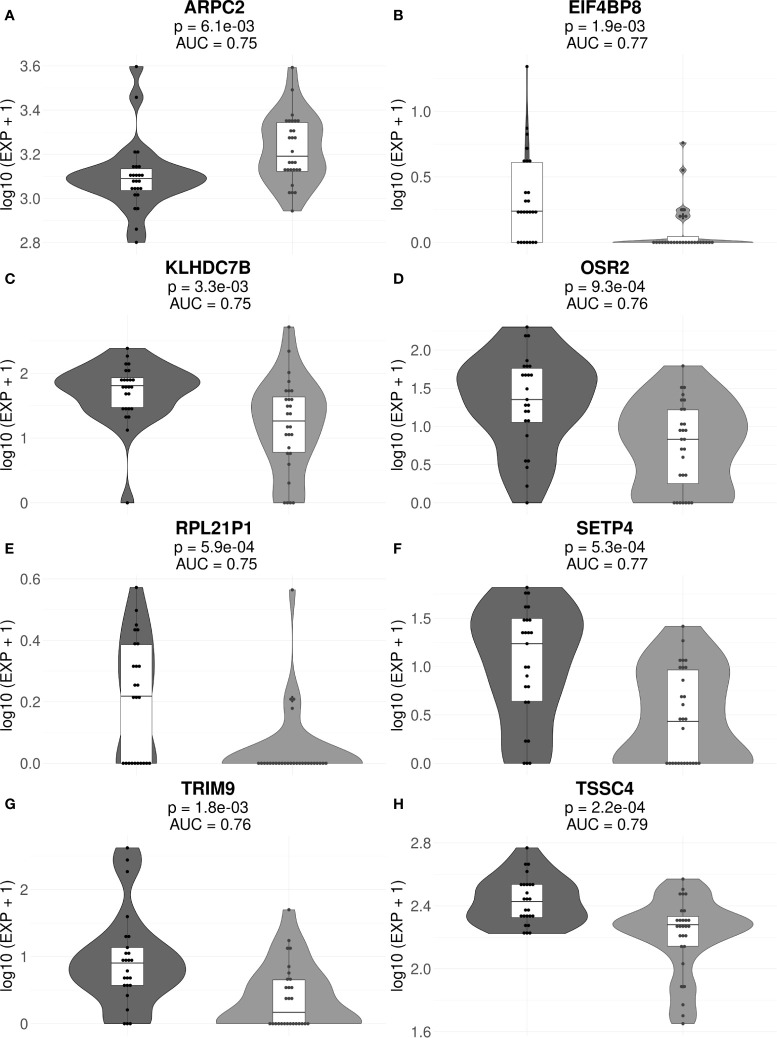
Gene expression levels of genes *ARPC2* **(A)**, *EIF4BP8* **(B)**, *KLHDC7B* **(C)**, *OSR2* **(D)**, *RPL21P1* **(E)**, *SETP4* **(F)**, *TRIM9* **(G)**, and *TSSC4* **(H)** in the full cohorts of MM responders and poor responders to PAD/VCD therapy. For every gene, paired t-test p-values and AUC values are shown. Each dot on the graph represents single MM sample. Grey indicates good treatment responders, black—poor responders.

**Figure 4:**
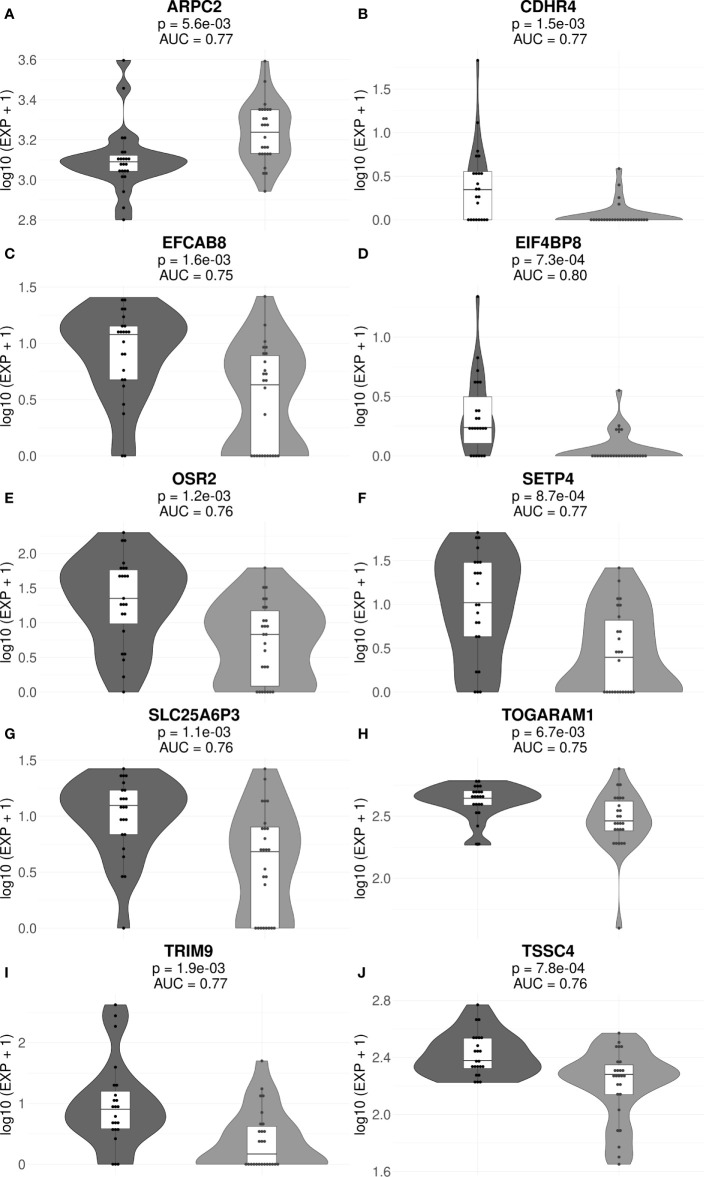
Gene expression levels of genes *ARPC2* **(A)**, *CDHR4* **(B)**, *EFCAB8* **(C)**, *EIF4BP8* **(D)**, *OSR2* **(E)**, *SETP4* **(F)**, *SLC25A6P3* **(G)**, *TOGARAM1* **(H)**, *TRIM9* **(I)**, and *TSSC4* **(J)** in the cohorts of MM responders and poor responders to VCD therapy. For every gene, paired t-test p-values and AUC values are shown. Each dot on the graph represents single MM sample. Grey indicates good treatment responders, black—poor responders.

The final list of core marker genes was then obtained by intersecting top 30 gene sets for all 53/47 sub-datasets. By using this procedure, we obtained a set of 8 core marker genes whose expression was characteristic for the MM patient (non)responder cohort (**Figures 3**, **4**; **Table 2**, **Supplementary Tables 3**, **4**).

**Table 2:** Core marker genes for the current PAD/VCD MM dataset (full cohort/VCD cohort).

Gene ID^1^	Cohort	Regulation in responders/non-responders^2^	Molecular function^3^
*ARPC2*	Full, VCD	Up/down	control of actin polymerization
*CDHR4*	VCD	Down/up	cell adhesion protein; sorting of heterogeneous cell types
*EFCAB8*	VCD	Down/up	Calcium ion binding
*EIF4BP8*	Full, VCD	Down/up	eukaryotic translation initiation factor 4B pseudogene 8
*KLHDC7B*	Full	Down/up	Kelch domain-containing protein 7B
*OSR2*	Full, VCD	Down/up	transcription factor
*RPL21P1*	Full	Down/up	ribosomal protein L21 pseudogene 1
*SETP4*	Full, VCD	Down/up	SET pseudogene 4
*SLC25A6P3*	VCD	Down/up	mitochondrial carrier; adenine nucleotide translocator, member 6 pseudogene 3
*TOGARAM1*	VCD	Down/up	microtubule binding
*TRIM9*	Full, VCD	Down/up	includes TRIM motif with three zinc-binding domains, a RING, a B-box type 1 and a B-box type 2, and a coiled-coil region
*TSSC4*	Full, VCD	Down/up	tumor suppressing subtransferable candidate 4

^1^Gene name according to HGNC nomenclature (63).

^2^Up/downregulation of marker genes in the treatment responders and non-responders, accordingly.

^3^Gene function according to GeneCards knowledgebase (64).

Interestingly, many of those genes were previously reported as cancer biomarkers. For example, gene *ARPC2* is prognostic biomarker in ovarian carcinomas (65). Gene *KLHDC7B* is regulated by interferon signaling pathway (66) and was previously published as the methylation marker in breast cancer (67) and also poor prognosis biomarker in triple negative breast cancer (68) and laryngeal cancer (69). *OSR2* gene is methylation marker in gastric cancer (70) and *TRIM9* was reported as cell-free DNA methylation marker of metastatic breast cancer (71). Finally, *TSSC4* gene is located in *11p15.5* locus, an important tumor-suppressor gene region which alterations are linked with the Beckwith–Wiedemann syndrome, Wilms tumor, rhabdomyosarcoma, adrenocortical carcinoma, and lung, ovarian, and breast cancer (https://www.genecards.org/cgi-bin/carddisp.pl?gene=TSSC4).

To improve performance of ML, we used a recent data preprocessing/trimming technique termed floating-window projective separator (FloWPS). This method increases AUC for most of ML methods in most of the clinically annotated gene expression datasets investigated (26, 48, 49). FloWPS improves the classifier robustness by performing dynamic data trimming and selecting sample-specific sets of relevant genes to prevent extrapolation in the feature space (described in detail in **Supplementary Text 1**). It prevents extrapolation in the feature space by excluding the features that cause such extrapolation. Second, it selects only *k* nearest neighbors for the training dataset to build a ML model similarly to the kNN method (72) to avoid confusing interference from too distant points from the training dataset in the feature space.

**Figure 5:**
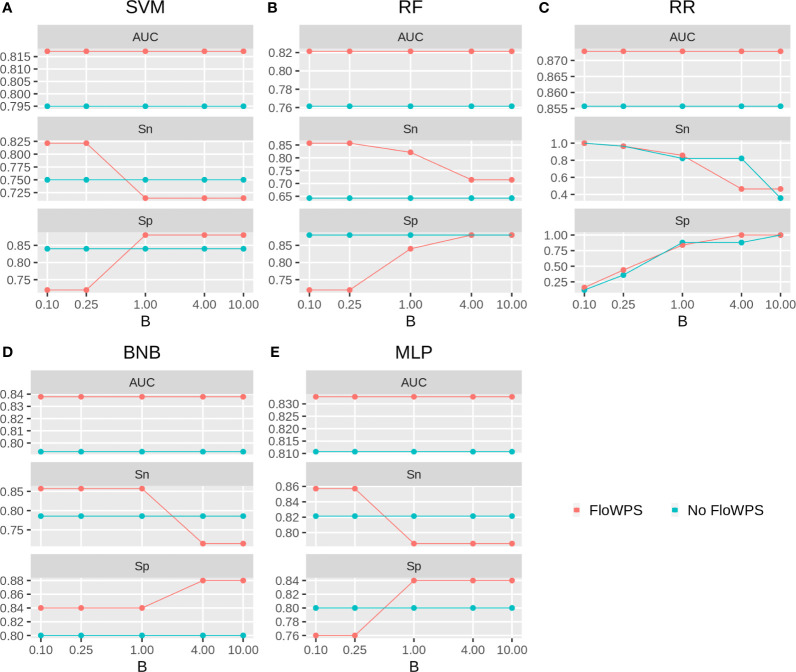
Area under receiver-operator curve (AUC), sensitivity (Sn) and Specificity (Sp) for five ML methods **(A)** linear SVM, **(B)** RF, **(C)** RR, **(D)** BNB, **(E)** MLP during classification of response to PAD/VCD treatment of 53 MM patients (full cohort). Parameter B is false positive vs. false negative balance factor.

**Figure 6:**
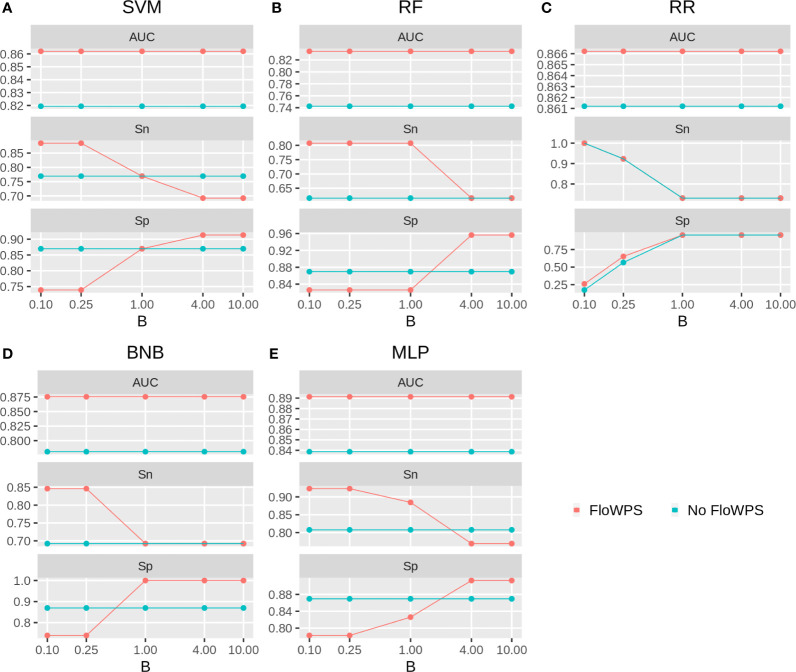
Area under receiver-operator curve (AUC), sensitivity (Sn) and Specificity (Sp) for five ML methods **(A)** linear SVM, **(B)** RF, **(C)** RR, **(D)** BNB, **(E)** MLP during classification of response to VCD treatment of 47 MM patients (VCD cohort). Parameter B is false positive vs. false negative balance factor.

We then built binary classifiers of MM response on PAD and VCD regimens using five ML methods: linear support vectors machine (SVM) (50, 73, 74), random forests (RF) (75), ridge regression (RR) (76), binomial naïve Bayes (BNB) (77), and multi-layer perceptron (MLP) (52, 74, 78). We checked performance of these methods with and without FloWPS. Cross-validation of the results for every method was done using the leave-one-out approach to calculate quality metrics such as AUC, sensitivity and specificity. The results are shown in **Figures 5**, **6** depending on different values of *B*, a relative balance factor for false positive and false negative errors. For all ML methods, application of FloWPS increased quality of the classifiers built as reflected by AUC metric (**Figures 5**, **6**). Taking together the three criteria of AUC, sensitivity (Sn) and specificity (Sp), the optimal solution was provided by the BNB method with FloWPS (AUC = 0.84) for the full cohort, and by MLP method with FloWPS (AUC = 0.89) for the VCD cohort.

### Comparison With Other Publicly Available Gene Expression Datasets With Known MM Response to Bortezomib-Containing Chemotherapy Regimens

We found seven publicly available MM datasets containing gene expression profiles annotated by clinical responses to the different bortezomib containing treatment schemes (25, 27–29, 32) summarized here on **Table 3**. Among them, only two chemotherapy scheme (bortezomib + thalidomide + dexamethasone) and PAD are currently accepted by the NCCN guidelines (79). All those alternative datasets were obtained by using expression microarrays whereas RNA sequencing that can be considered gold standard of cancer transcriptomic analyses (80) was used here for the first time to characterize PAD and VCD treatment efficiencies.

**Table 3:** General characteristics of bortezomib chemotherapy response-annotated MM datasets.

Reference	Dataset ID	Therapy	Experimental platform	Number *N* of cases (R vs NR)	Number of *core marker genes*
	Current study (full cohort)	Bortezomib, doxorubicin, dexamethasone (PAD) AND/OR bortezomib, cyclophosphamide, dexamethasone (VCD)	RNA sequencing, Illumina HiSeq 3000	53 (28 R: *complete response* + *very good partial response*; 25 NR: *partial response* + *minimal* response)	8
	Current_Study_VCD	Bortezomib, cyclophosphamide, dexamethasone (VCD)	RNA sequencing, Illumina HiSeq 3000	47 (24 R: *complete response* + *very good partial response*; 23 NR: *partial response* + *minimal* response)	10
(25)	GSE9782	Bortezomib monotherapy	Affymetrix Human Genome U133 Array	169 (85 R: *complete response* + *partial response*; 84 NR: *no change* + *progressive disease*)	18
(29)	GSE68871	Bortezomib-thalidomide-dexamethasone	Affymetrix Human Genome U133 Plus	118 (69 R: *complete*, *near-complete* and *very good partial responders*; 49 NR: *partial*, *minor* and *worse*)	12
(27)	GSE55145	Bortezomib followed by ASCT	Affymetrix Human Exon 1.0 ST Array	61 (33 R: *complete*, *near-complete* and *very good partial responders*; 28 R: *partial*, *minor* and *worse*)	14
(32, 41)	GSE19784_1	Bortezomib, doxorubicin, dexamethasone (PAD)	Affymetrix Human Genome U133 Plus 2.0 Array	61 with ISS stage I [32 R, 29 NR (32)]	7
(32, 41)	GSE19784_2	Bortezomib, doxorubicin, dexamethasone (PAD)	Affymetrix Human Genome U133 Plus 2.0 Array	51 with ISS stage II [33 R, 18 NR (32)]	12
(32, 41)	GSE19784_3	Bortezomib, doxorubicin, dexamethasone (PAD)	Affymetrix Human Genome U133 Plus 2.0 Array	41 with ISS stage III [29 R, 12 NR (32)]	11
(32, 34)	GSE2658	Bortezomib, doxorubicin, dexamethasone (PAD)	Affymetrix Human Genome U133 Plus 2.0 Array	208 [55 R, 153 NR (32)]	16

When processed in the same way as the current experimental dataset to apply different ML methods, 7–18 core marker genes distinguishing good and poor responders were obtained for these literature datasets (26). We found no intersections between the core marker genes corresponding to these and current experimental datasets (**Table 4**). Moreover, using the current experimental set of 8/10 core marker genes (for the full/VCD cohorts, respectively) couldn’t be used for building robust classifiers with the same repertoire of ML methods (data not shown). This can be due to differences in both gene expression interrogation methods, MM heterogeneity, and the composition of MM treatment schemes. Similarly, findings of *Intergroupe Francophone du Myélome* (IFM) suggest the absence of a robust gene signature associated with the treatment response (14, 81, 82).

**Table 4:** Core marker genes identified for bortezomib chemotherapy response-annotated MM datasets; genes that are overexpressed in the treatment responders are marked by (+), downregulated in the responders by (−).

Current study full cohort	Current study VCD cohort	GSE9782	GSE68871	GSE55145	GSE19784_1	GSE19784_2	GSE19784_3	GSE2658
ARPC2 (+)	ARPC2 (+)	ABHD14A (−)	BORCS8 (-)	AKNA (−)	ACE2 (+)	DCUN1D2 (−)	ANKRD11 (−)	BCOR (+)
EIF4BP8 (−)	CDHR4 (−)	ATP2B4 (+)1	BTG1 (-)	CATSPER3 (+)	C22orf24 (−)	GTF2H5 (+)	FN1 (+)	CENPE (+)
KLHDC7B (−)	EFCAB8 (−)	ATP5S (−)	CCND1 (-)	EMP3 (+)	FAM132A (−)	HAUS8 (−)	HDAC2 (−)	COX6C (+)
OSR2 (−)	EIF4BP8 (−)	ATP5SL (−)	CSTB (−)	MYH9 (−)	GPR124 (+)	PPIEL (−)	HS3ST5 (+)	FH (+)
RPL21P1 (−)	OSR2 (−)	ATP6V0D1 (+)	CTCFL (+)	NDRG2 (+)	GTF2A1L (+)	PSPC1 (−)	KRT35 (−)	FUNDC1 (+)
SETP4 (−)	SETP4 (−)	B2M (+)	GAS6_AS1 (−)	NUCB2 (+)	PPP4R4 (+)	RAD52 (−)	LINC00511 (−)	G2E3 (+)
TRIM9 (−)	SLC25A6P3 (−)	BCL2L11 (−)	NOMO3 (+)	PASK (−)	RP11.680G24.5 (−)	RBP5 (+)	MFSD4 (−)	HMGB3 (+)
TSSC4 (−)	TOGARAM1 (−)	C7orf26 (−)	ORAI1 (−)	RAPGEF1 (−)		RP5.1098D14.1 (−)	NOXO1 (−)	HMGB3P1 (+)
	TRIM9 (−)	CCNB1IP1 (−)	PLOD3 (+)	RRP7BP (−)		SMARCA2 (+)	REM1 (−)	MEI1 (−)
	TSSC4 (−)	COX7C (−)	SCN9A (+)	TMEM131L (−)		STK32A (−)	RP11.960B9.2 (−)	NEDD9 (−)
		DLST (−)	STK33 (+)	TMEM99 (+)		TIAM1 (−)	SNCAIP (−)	PAM (−)
		DLSTP1 (−)	SYBU (+)	TRAF3 (−)		TMEM57 (+)		RP11.164P12.4 (−)
		FAM106A (+)		TRAF4 (−)				S100PBP (+)
		GFER (−)		ZNF286A (+)				SHCBP1 (+)
		NDUFB1 (-)						SMC4 (+)
		PATZ1 (−)						UNC13C (+)
		RPS7 (−)						
		TCP11L1 (−)						

However, for all the literature datasets investigated utilization of best ML methods enhanced by FloWPS using their own core biomarker genes resulted in high-quality classifiers with ROC AUC varying in the range 0.79–0.96 (**Table 5**). Interestingly, one of those previous MM datasets (25) for bortezomib monotherapy (best AUC = 0.8) was previously characterized as “inconvenient” for ML because other attempts to build a response classifier without using core marker gene approach and FloWPS were unsuccessful resulting in AUC <0.66 (83–87).

**Table 5:** Best ROC AUC and AUPR (precision-recall AUC) values obtained for good versus poor responder classifiers built using different ML methods without/with FloWPS for different MM annotated expression datasets.

Dataset	SVM	RF	RR	BNB	MLP
**Current study full ROC AUC**	0.80/0.82	0.76/0.82	0.86/0.87	0.79/0.84	0.81/0.83
**Current study full AUPR**	0.79/0.82	0.78/0.79	0.88/0.90	0.78/0.83	0.79/0.81
**Current study VCD ROC AUC**	0.82/0.86	0.74/0.83	0.86/0.87	0.78/0.88	0.84/0.89
**Current study VCD AUPR**	0.82/0.86	0.76/0.86	0.86/0.84	0.79/0.92	0.83/0.88
**GSE9782 ROC AUC**	0.68/0.72	0.68/0.80	0.77/0.77	0.73/0.76	0.72/0.76
**GSE9782 AUPR**	0.65/0.70	0.70/0.80	0.77/0.77	0.69/0.76	0.69/0.74
**GSE68871 ROC AUC**	0.68/0.77	0.73/0.83	0.78/0.77	0.74/0.84	0.70/0.80
**GSE68871 AUPR**	0.64/0.76	0.73/0.83	0.79/0.77	0.71/0.80	0.69/0.76
**GSE55145 ROC AUC**	0.78/0.82	0.77/0.90	0.87/0.84	0.82/0.87	0.80/0.85
**GSE55145 AUPR**	0.72/0.84	0.72/0.84	0.88/0.85	0.83/0.82	0.83/0.82
**GSE19784_1 ROC AUC**	0.65/0.82	0.74/0.77	0.84/0.84	0.74/0.84	0.72/0.81
**GSE19784_1 AUPR**	0.64/0.77	0.71/0.77	0.86/0.84	0.72/0.84	0.69/0.79
**GSE19784_2 ROC AUC**	0.83/0.87	0.75/0.82	0.92/0.94	0.88/0.94	0.86/0.87
**GSE19784_2 AUPR**	0.85/0.91	0.79/0.86	0.96/0.97	0.92/0.97	0.88/0.89
**GSE19784_3 ROC AUC**	0.84/0.94	0.84/0.86	0.95/0.95	0.86/0.96	0.91/0.94
**GSE19784_3 AUPR**	0.89/0.95	0.89/0.90	0.98/0.98	0.92/0.98	0.95/0.96
**GSE2658 ROC AUC**	0.72/0.77	0.67/0.79	0.79/0.79	0.76/0.78	0.63/0.72
**GSE2658 AUPR**	0.45/0.55	0.51/0.61	0.58/0.61	0.49/0.54	0.42/0.48

For ROC AUC metric, FloWPS enhancement was beneficial for all global ML methods such as SVM, RF, BNB, and MLP. Likewise, it increased the precision-recall AUC (AUPR) metric for global ML methods in most datasets (**Table 5**). This was also in line with the previous findings where it could improve accuracy and Matthews correlation coefficient metrics (48).

### Differentially Expressed Gene Analysis

We performed the analysis for differentially expressed genes that distinguish responders from non-responders using the DESeq2 (51) method with the criteria *pAdjusted <*0.05, |LFC_2_| >0.5 (**Supplementary Figure 2**). Interestingly, we found no marker role of bortezomib molecular target genes *PSMB1* and *PSMB5* for neither dataset, as reflected by AUC levels of less than 0.7 (**Supplementary Figure 1**).

**Figure 7:**
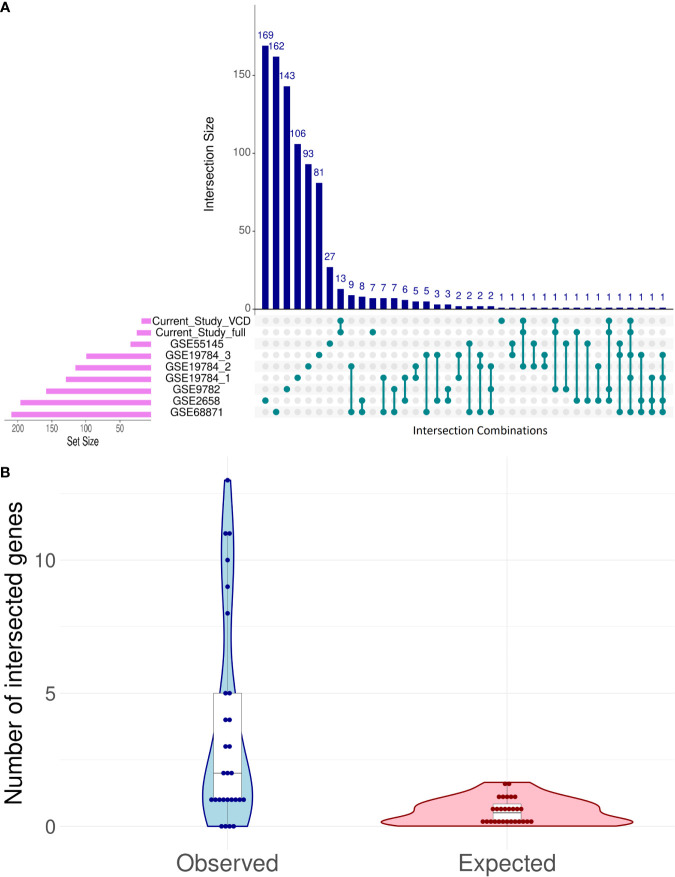
Intersection analysis for differentially expressed genes (DEG) distinguishing good and poor treatment responders in eight bortezomib MM datasets **(A)**. *Observed* vs *expected* (under the hypothesis of random DEG distribution) numbers of intersection events in all possible pairwise comparisons **(B)**.

Despite the lack of intersection between core marker genes that served for ML model creation, there were several differential genes that were regulated similarly among the good vs poor responders in the different datasets, and the intersection pattern was not random (**Figure 7**).

The intersections between differential genes for all datasets were analyzed using UpSetR method (88); **Figure 7A**. To assess randomness of differential genes in the MM datasets, we used the following test. Differential gene sets for all MM datasets can form 7·8/2 = 28 pair intersections. For each of these paired intersections the number of *observed* intersected genes was calculated and compared with the random expectation model (**Figure 7B**). For random expectations, in each dataset we picked 1,000 times randomly the observed number of differential genes, and modeled all 28 possible intersections (**Figure 7B**).

The maximum similarities were observed between the datasets GSE68871 and GSE19784_1 (Jaccard coefficient *J* = 0.042), GSE68871 and GSE19784_2 (*J* = 0.037), GSE68871 and GSE1978_4 (*J* = 0.030), and GSE68871 and GSE2658 (*J* = 0.028); **Supplementary Table 5**.

Interestingly, we found several common differential genes in the current experimental RNAseq and in the previous microarray datasets (**Table 6**). All these common differential genes were overexpressed in the good vs poor treatment responders. Among them, the fibroblast growth factor receptor 3 gene (*FGFR3*) was found three times, and the transcription factor MAF gene was detected twice. Other differential genes (*IGHA2*, *IGHV1-69*, *GRB14*) were detected once. Among them, *GRB14* was found only for the full (PAD+VCD) dataset, but not in the reduced VCD dataset. Other abovementioned differential genes were shared for the PAD + VCD and VCD datasets.

**Table 6:** Common differentially expressed genes (DEGs) in the current experimental dataset (full or VCD only cohorts) and in seven previously published MM datasets.

Dataset	GSE9782	GSE68871	GSE55145	GSE19784_1	GSE19784_2	GSE19784_3	GSE2658
Common DEGs with full cohort	*IGHA2 (+), MAF (+)*	*FGFR3 (+)*	***–***	*FGFR3 (+)*	*IGHV1-69 (+)*	*FGFR3 (+)*	*GRB14 (+), MAF (+)*
Common DEGs with VCD cohort	*IGHA2 (+), MAF (+)*	*FGFR3 (+)*	***–***	*FGFR3 (+)*	*IGHV1-69 (+)*	*FGFR3 (+)*	*MAF (+)*

(+) marks genes overexpressed in the good treatment responders.

Four out of these five differential genes (*FGFR3, MAF, IGHA2, IGHV1-69*) are associated with translocation of immunoglobulin locus on 14q32 region that frequently occurs in MM (89), which clearly connects our results with the MM biology. Differential genes *IGHA2* and *IGHV1-69* are both located on the above 14q32 locus and encode for immunoglobulin heavy chain constant region alpha 2, and immunoglobulin heavy chain variable regions 1–69, respectively. To our knowledge, they were never associated before with bortezomib effectiveness in MM and in other tumors. We also found no known associations for *GRB14* with MM.

Our results on *FGFR3* are congruent with the previous findings. Fibroblast growth factor receptor 3 (FGFR3) is receptor tyrosine kinase which prevents apoptosis in MM cells and promotes adhesion to bone marrow stromal cells (90). It is overexpressed in ~20% of MM cases (91). High expression of *FGFR3* was reported as the positive clinical response prognostic factor for bortezomib monotherapy (92), and for the bortezomib + thalidomide + dexamethasone (VTD) regimen (93). In parallel with *FGFR3* activating mutations (94), it was also shown a factor mediating and positively correlating with bortezomib-related apoptosis in cultured MM (91) and lymphoma (95) cells. Interestingly, at the same time *FGFR3* overexpression was reported as a negative factor for treatment with thalidomide, another targeted MM therapeutic (96).

However, for transcriptional factor MAF contradictory reports have been published that its expression is either positive (97), neutral (98–100) or negative (101) prognostic factor for response on bortezomib containing treatments. MAF is a transcriptional activator of many genes, including cyclinD2 and Integrin-β7 (102). Translocation of MAF into immunoglobulin locus is initiating oncogenic event in 5–10% of MM cases, and it was estimated to be up-regulated in 40–50% of all multiple myelomas (103, 104).

To further functionally characterize the differential gene sets, we performed Gene Ontology (GO) analysis (105), **Supplementary Figure 3**. We identified enrichment clusters only for four datasets investigated: for the current study, GSE9782, GSE19784_1, and GSE2658. Those clusters corresponded predominantly to the various immune cell-specific processes (**Supplementary Figure 3**).

We also considered 20 experimental MM cases treated with PAD regimen and found four differential genes between the good and poor responders (**Supplementary Figure 2C**), including gene *SEZ6L2* which was common with the literature dataset GSE9782. We found no previous mentions of the association of this gene with MM.

### Gene Expression Changes in MM After PAD/VCD *T*reatment

To our knowledge, MM gene expression profiles before and after relapse on PAD/VCD regimens had never been reported in the literature. For two MM patients included in this study, we were able to isolate CD138+ fraction of MM cells for the bone marrow biopsies taken after recurrence of the disease (**Table 7**). The patient 111 sequentially had four courses of first-line PAD and two courses of VCD chemotherapy regimens and showed partial response before relapse. In turn, the patient 115 also had four courses of first-line PAD and two courses of VCD chemotherapy regimens but demonstrated only minimal response before relapse (**Supplementary Table 1**).

**Table 7:** Normalized expression levels of bortezomib targeting genes in MM patients before and after PAD/VCD treatment.

Patient ID	Best response status	Sample status	*PSMB1* expression^1^	*PSMB5* expression^1^
MM111	Partial response	Pretreatment	880,4	669,9
		Relapse	553	483,5
MM115	Minimal response	Pretreatment	814	456,3
		Relapse	604,8	282,8

^1^Normalized counts of gene-mapped RNA sequencing reads.

We compared expressions of bortezomib targeted genes in those patient biosamples before and after PAD/VCD treatment (**Table 7**). Interestingly, genes for both molecular targets of bortezomib (*PSMB1, PSMB5*) were downregulated after PAD/VCD treatment in both patients. This can represent tumor adaptation to the chemotherapy regimens used. However, it should be mentioned that those genes couldn’t serve as the bortezomib response prognostic biomarkers in all datasets investigated here (**Supplementary Figure 1**).

## Data Availability Statement

The datasets presented in this study can be found in online repositories. The names of the repository/repositories and accession number(s) can be found in the article/**Supplementary Material**.

## Ethics Statement

The studies involving human participants were reviewed and approved by the ethical committees of the Sechenov Moscow First Medical University, of the Clinical Center Vitamed (Moscow), and of the National Research Center for Hematology (Moscow, Russia). The patients/participants provided their written informed consent to participate in this study.

## Author Contributions

NB, MSu, VS, AS, MN, NG, MSo, and AB contributed conception and design of the study. NG performed autopsies. MN, LM, and AS isolated and prepared blood tissue samples. MSu and XL performed molecular analyses. NB, MR, MSu, NG, MSo, AGu, AGa, VT, and AB analyzed the data. NB, MR, MSo, VS, and AB wrote the paper. All authors contributed to the article and approved the submitted version.

## Funding

The study was supported by the Oncobox research program in oncology, by OmicsWay Corp., by Amazon and Microsoft Azure grants for cloud-based computational facilities, by Russian Foundation for Basic Research grant 19-29-01108 to AB (algorithmic implementation) and by Russian Scientific Foundation grant to MS 20-75-10071 (cancer RNA sequencing). The funder bodies were not involved in the study design, collection, analysis, interpretation of data, the writing of this article or the decision to submit it for publication.

## Conflict of Interest

The AG, VT and MS were employed by Omicsway Corp. and Oncobox Ltd. AB was employed by Omicsway Corp.

The remaining authors declare that the research was conducted in the absence of any commercial or financial relationships that could be construed as a potential conflict of interest.

## Supplementary Material

Supplementary Table 1 Clinical information.

Supplementary Table 2 Quality control metrics.

Supplementary Table 3 Core marker genes for full MM cohort.

Supplementary Table 4 Core marker genes for VCD MM cohort.

Supplementary Table 5 Jaccard similarity, observed and expected (under the hypothesis of random DEG distribution) number of common DEGs between different pairs of datasets.

Supplementary Text 1 FloWPS data trimming method.

Supplementary Figure 1 Gene expression levels of target bortezomib genes *PSMB1* and *PSMB5.* Gene expression levels in the cohorts of MM responders and poor responders to therapy. For every gene, paired t-test p-values and AUC values are shown. Each dot on the graph represents single MM sample. Grey indicates good treatment responders, black – poor responders.

Supplementary Figure 2 Volcano pots for DEGs distinguishing good and poor responders in eight MM datasets: Current Study full **(A)** Current Study VCD **(B)** Current Study PAD **(C)** GSE9782 **(D)** GSE68871 **(E)** GSE55145 **(F)** GSE19784_1 **(G)** GSE19784_3 **(H)** GSE19784_3 **(I)** GSE2658 **(J)**.

Supplementary Figure 3 Gene Ontology enrichment analysis using the *clusterProfile* package (105) for the following datasets: Current Study full **(A)** Current Study VCD **(B)** GSE9782 **(C)** GSE19784_1 **(D)** and GSE2658 **(E)**. Other datasets had no GO terms enriched with DEGs.
