# Supplementary material for: Machine Learning Applicability for Classification of PAD/VCD Chemotherapy Response Using 53 Multiple Myeloma RNA Sequencing Profiles
Source: Front Oncol. 2021 Apr 15;11:652063. doi: 10.3389/fonc.2021.652063 (PMC8083158; doi:10.3389/fonc.2021.652063)
Supplement: Supplementary Text 1 — FloWPS data trimming method. [file Table_1.docx]

**Supplementary Text 1: detailed description of FloWPS data trimming method**

Imagine (see also (Borisov and Buzdin, 2019; Tkachev et al., 2019, 2020)) that we have to classify the clinical response for a certain patient *i* (called *patient of interest)* from a given dataset of expression/mutation profiles linked to known (positive or negative) clinical responses. Let the whole dataset contain *N* patients so that the remaining *N*-1patients form the *preceding dataset* *D_i_*, for the patient of interest. For ML *without data trimming*, in the feature space **F** = (*f*_1_,…, *f_S_*) all *N*-1 remaining patients are used to build the classifier. But in the case of FloWPS, LOO procedure will be applied to classify every sample *j ≠ i* from the preceding dataset *D_i_* without sample *i*, and *N*-2 remaining samples may be used for such a classification of sample *j*. To avoid extrapolation in the feature space, we consider the subset **F***_ij_* of *relevant features* (Borisov and Buzdin, 2019; Tkachev et al., 2019, 2020). A feature *f_s_* is considered relevant for the sample *j* if on its axis there are at least *m* projections from *N*-2 training samples, which are larger than *f_s_*(*i,j*), and, at the same time, at least *m*, which are smaller than *f_s_*(*i,j*), when *m* is a non-negative integer parameter (Fig. S2_1 A). The maximum possible *m* value is (*N*-2)/2, since if *m* is less than (*N*-2)/2, then no relevant features may be chosen. Similarly, the minimal case of *m* = 0 also corresponds to no feature selection. Note that the resulting subset of relevant features **F***_ij_*(*m*) will be individual for every pair of samples *i* and *j* (Borisov and Buzdin, 2019; Tkachev et al., 2019, 2020).

Moreover, in the space **F***_ij_* (*m*) only *k* closest samples to sample *j* will be allowed for training among the remaining (*N*-2) cases. As a measure for proximity, the Euclidean distance is used (Tkachev et al., 2019). Here *k* is another integer parameter that specifies the number of nearest neighbors in the subspace of selected features (Fig. S2_1 B). The maximal possible *k* is *N*-2, which corresponds to no training sample selection. In contrast, when *k* is too low, there is an increased risk of ML error, due to presence of too small number of training points among the *k* nearest neighbors (Fig.S2_1 B).


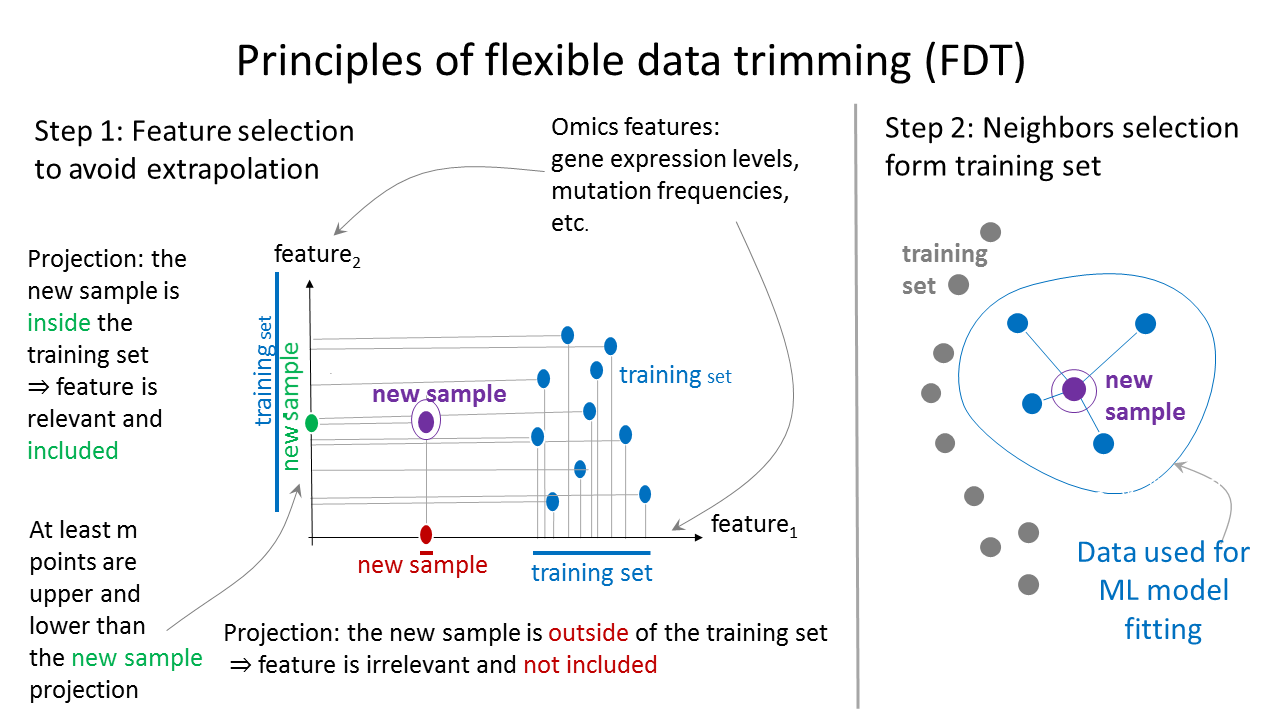

Fig. S2_1 Outline of FLOating Window Projective Separator (FloWPS) approach. Selection of relevant features (A) and nearest neighbors (B) are schematized.

After selection of relevant features and nearest neighbors for the sample *j*, the ML model is trained using nearest neighbors only, and used for prediction of a clinical response, *P_ij_*(*m*,*k*), for the patient *j*. After repeating this procedure for all other *j* ≠ *i*, we obtain the area-under the ROC curve, AUC*_i_*(*m*,*k*), for all but *i*-th samples for fixed values of data trimming parameters *m* and *k*.

The AUC*_i_*(*m*,*k*) can be then analyzed as a function of *m* and *k* (Tkachev et al., 2019).

Over the range of possible *m* and *k* values, we compare the AUC*_i_* function (Tkachev et al., 2019). All pairs of (*m*,*k*) values that provide AUC*_i_*(*m*,*k*) > *p*·max(AUC*_i_*(*m*,*k*)) form the prediction-accountable set *S_i_* for the patient of interest *i* (Borisov and Buzdin, 2019; Tkachev et al., 2019, 2020), where *p* is the confidence threshold, which could vary from 0.90 till 0.95 in our previous computational experiments (Tkachev et al., 2019).

Finally, the FloWPS prediction *P_Fi_* for the sample of interest *i*, is calculated by averaging the ML predictions over the prediction-accountable set *S_i_*: $P_{Fi}={mean}_{Si}\left( P_{i}\left( m,k \right) \right)$. By repeating this procedure for all other samples, a set of FloWPS predictions will be obtained for the whole dataset (Tkachev et al., 2019, 2020).

The overview of LOO cross-validation algorithm for the expression-based classifier of clinical response is shown in Fig. S2_2.


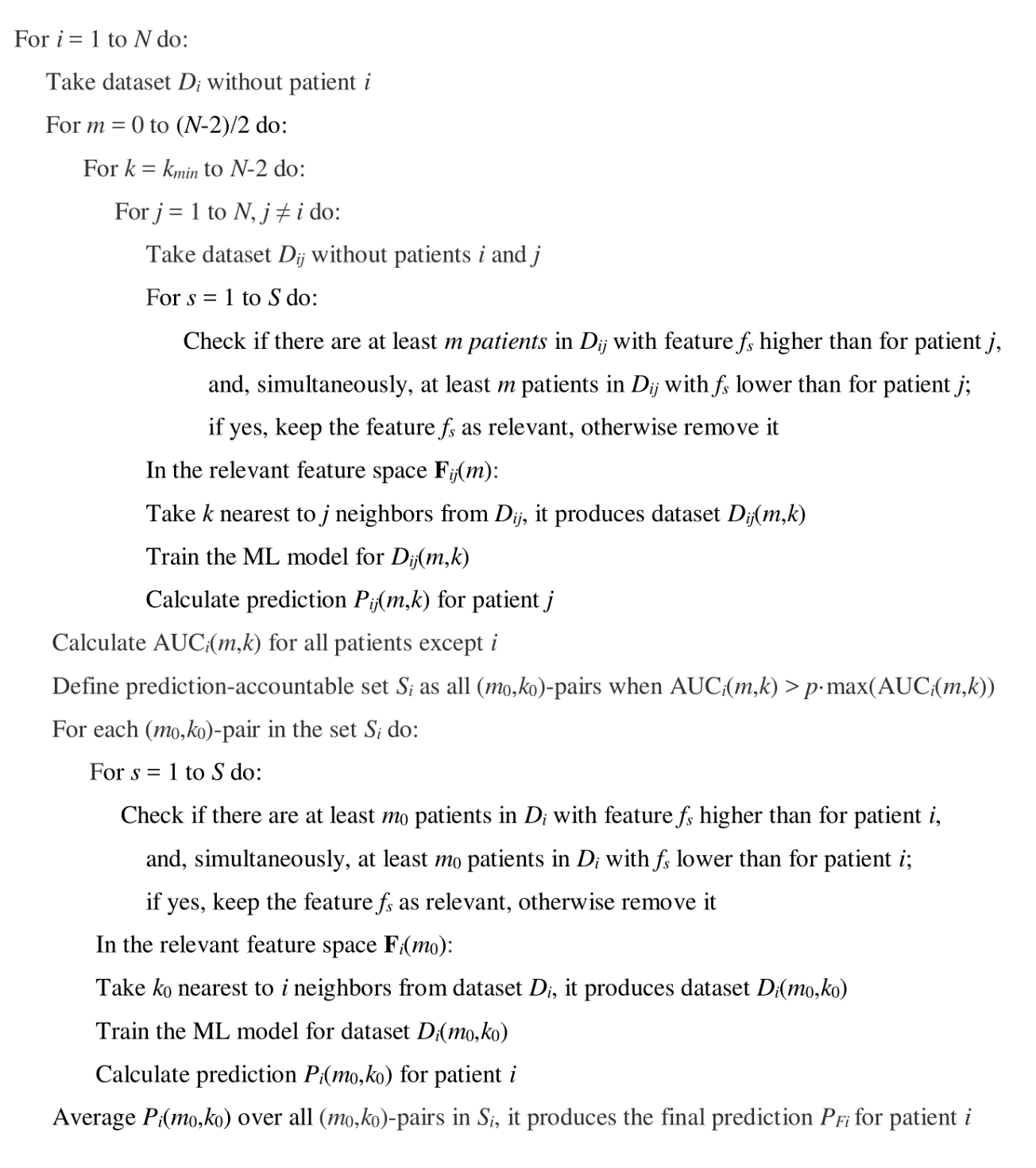


Fig. S2_2. The algorithm of data trimming used for LOO cross-validation of the clinically annotated gene expression datasets. Indexes *i* and *j* denote samples (patients), index *s* denotes pairs of (*m*_0_,*k*_0_)-values in the prediction-accountable set, and indexes *m* and *k* denote the data trimming parameters.

**References**

Borisov, N., and Buzdin, A. (2019). New Paradigm of Machine Learning (ML) in Personalized Oncology: Data Trimming for Squeezing More Biomarkers From Clinical Datasets. Front. Oncol. *9*, 658.

Tkachev, V., Sorokin, M., Mescheryakov, A., Simonov, A., Garazha, A., Buzdin, A., Muchnik, I., and Borisov, N. (2019). FLOating-Window Projective Separator (FloWPS): A Data Trimming Tool for Support Vector Machines (SVM) to Improve Robustness of the Classifier. Front. Genet. *9,* 717.

Tkachev, V., Sorokin, M., Borisov, C., Garazha, A., Buzdin, A., and Borisov, N. (2020). Flexible Data Trimming Improves Performance of Global Machine Learning Methods in Omics-Based Personalized Oncology. Int. J. Mol. Sci. *21*, 713.
